# Neuronal intranuclear inclusion disease with mental abnormality: a case report

**DOI:** 10.1186/s12883-020-01933-8

**Published:** 2020-09-23

**Authors:** Xiaosa Chi, Man Li, Ting Huang, Kangyong Tong, Hongyi Xing, Jixiang Chen

**Affiliations:** 1 Department of Neurology, Union Hospital, Tongji Medical College, Huazhong University of Science and Technology, Wuhan, 430022 China; 2 Department of Neurology, The First People’s Hospital of Zaoyang, Zaoyang, 441299 China

**Keywords:** Neuronal intranuclear inclusion disease, Mental abnormality, Skin biopsy, Intranuclear inclusion

## Abstract

**Background:**

Neuronal intranuclear inclusion disease (NIID) is a chronic progressive neurodegenerative disease that is characterized by the discovery of eosinophilic hyaline intranuclear inclusions in the central and peripheral nervous systems and visceral organs. In this paper, we report a case of an adult-onset neuronal intranuclear inclusion disease presenting with mental abnormality in China.

**Case presentation:**

A 62-year-old woman presented with mental abnormality and forgetfulness for 3 months before she was admitted to our hospital. There were prodromal symptoms of fever before she had the mental disorder. Encephalitis was first suspected, and the patient underwent lumbar puncture and brain magnetic resonance imaging (MRI). A cerebrospinal fluid (CSF) examination indicated normal pressure, a normal white blood cell count, and slightly elevated protein and glucose levels. Coxsackie B virus, enterovirus, and cytomegalovirus tests showed normal results. Bacterial culture and *Cryptococcus neoformans* test results were negative. The contrast-enhanced MRI of the brain was normal. The brain diffusion-weighted imaging (DWI) showed a symmetrically distributed strip-shaped hyperintensity signal of the corticomedullary junction in the bilateral frontal, parietal, and temporal lobes. We considered the diagnosis of the NIID, and therefore, skin biopsy was performed. The electron microscopy revealed that intranuclear inclusions in the nucleus of fibrocytes existed and were composed of filaments.

**Conclusions:**

NIID is a rare neurodegenerative disease with diverse clinical manifestations. In clinical work, when a patient presents with abnormal mental behavior and exhibits hyperintensity signals on DWI images of the corticomedullary junction, it is crucial to consider the diagnosis of NIID.

## Background

NIID is a rare progressive neurodegenerative disease characterized by eosinophilic hyaline intranuclear inclusions in the central nervous systems (CNS), peripheral cells of the autonomic nervous system, visceral organs, and the skin [1]. In 1968, the first case of NIID was reported by Lindenberg et al. [2]. NIID is pathologically characterized by the presence of ubiquitinated intranuclear inclusions in the neurons [3]. It is difficult to identify the disease, as it has highly variable clinical manifestations, for example, dementia, abnormal behavior, muscle weakness, cerebellar ataxia, parkinsonism, epileptic seizures, and autonomic impairment. Furthermore, it can be sporadic or familial, and the age of onset varies from infant to adult. It has been reported that skin biopsy is useful for the antemortem diagnosis [4]. Thus, an increasing number of cases of NIID were found [5]. The identification of the GGC repeat expansion at the 5′ end of *NOTCH2NLC as* the genetic cause of NIID will help in identifying the molecular pathogenesis in NIID [6]. Most of the cases were observed in Japan, but there were few case reports in China. We report a case of a sporadic adult-onset neuronal intranuclear inclusion disease with mental abnormality, and the NIID was diagnosed through skin biopsy and typical changes on MRI.

## Case presentation

A 62-year-old woman was admitted to our hospital because of mental abnormality and hypomnesia for 3 months. The main mental symptoms were gibberish, irrational talk, impaired mental attention, without apparent hallucinations and delusions. The patient did not present with fluctuating consciousness, lack of consciousness, or loss of consciousness. The family of the patient reported that she had a history of fever before she experienced the mental symptoms. Her highest body temperature was 38.6 °C. Her axillary temperature became normal after she received antibiotic therapy for 4 days. However, she gradually developed symptoms of mental abnormality. She also intermittently complained of abdominal pain, vomiting, constipation, and urinary incontinence for 1 month. She did not experience other discomforts, such as headaches, dizziness, and convulsion. She visited a local hospital in October 2018, and the routine blood test showed that the white blood cell counts were normal, and the percentage of lymphocytes was high. The blood potassium was 1.91 mmol/l (normal 3.5-5.3 mmol/l), and the prolactin level was 90.55 μg/l (normal 2.74–26.72 μg/l). The free thyroxine was slightly high, the thyroglobulin antibody was 12.32 IU/ml (normal 0–115 IU/ml), and the thyroid peroxidase antibody was 10.42 IU/ml (normal 0–34 IU/ml). The testing results of antinuclear antibody, anti-neutrophil cytoplasmic antibody, anti-Jo-1 antibody, anti-dsDNA antibody, anti-Scl-70 antibody, anti-SSA antibody, and anti-SSB antibody were yielded negative, and the tests for hepatitis, HIV, and syphilis virus were also negative. The local hospital suspected intracranial infection, endocrine disease, and electrolyte disorders and gave her symptomatic treatment. Nevertheless, her symptoms showed no noticeable improvement. It was difficult to diagnose the disease, so the patient was admitted to our hospital. The patient had a history of diabetes, hypertension, erosive gastritis, and cataract surgery, but she had no family history of neurological diseases.

The physical examination revealed that her body temperature was 36.8 °C, and there was tenderness below the xiphoid. The neurological examination showed impaired mental attention and reaction capacity, slow speech, normal manifestations of the brain nerve, normal muscle strength, and muscle tone, negative meningeal irritation sign and pathological reflex, normal tendon reflex. The patient could not cooperate with the sensory system examination. The mini-mental state examination (MMSE) score was only 6/30.

**Fig. 1:**
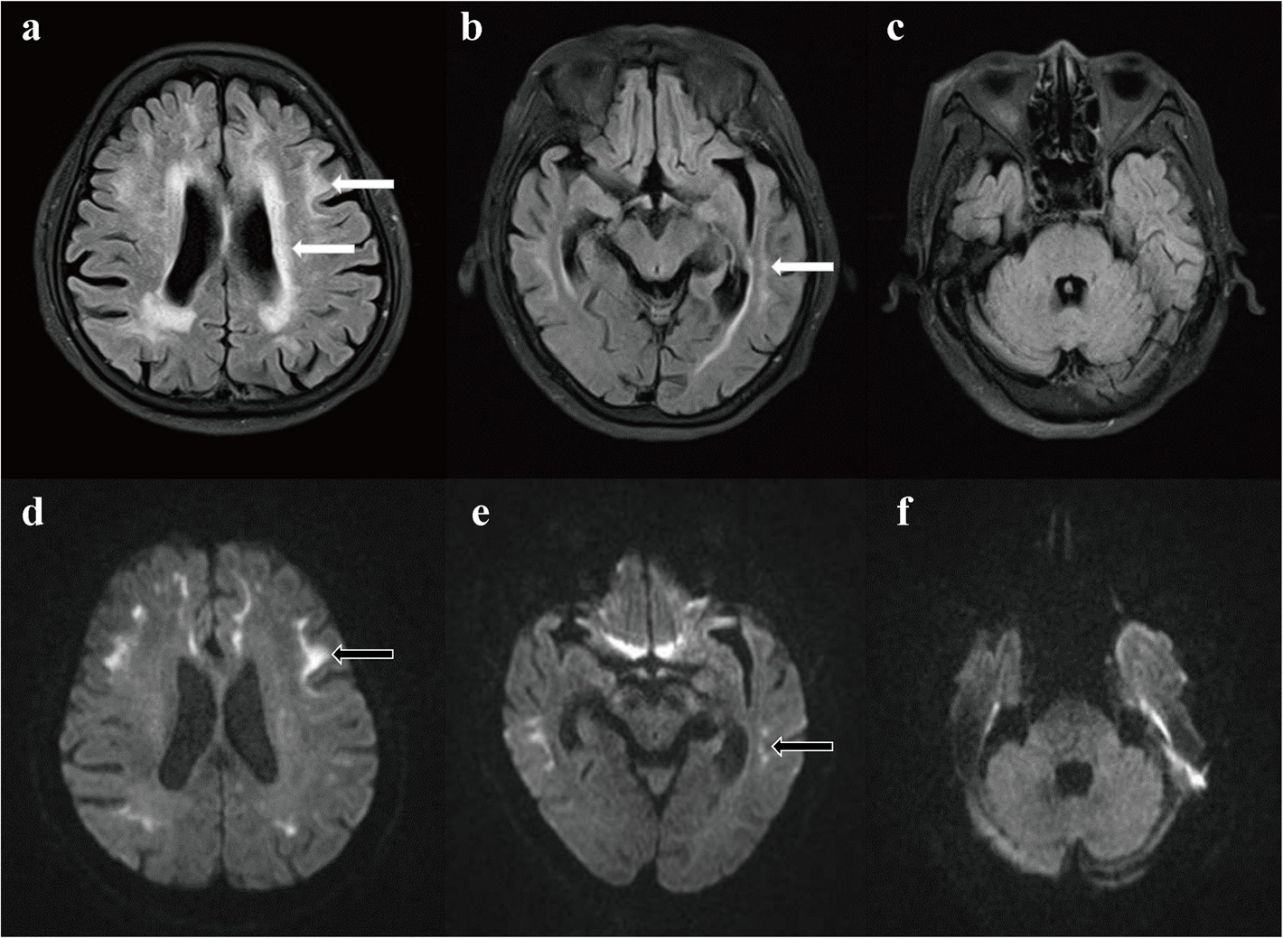
**a**-**f**: Cranial MRI performed 3 months after onset. **a**-**c**: Leukoencephalopathy was evident on the T2 FLAIR images. A high-signal intensity in the white matter of the cerebral hemisphere, especially at the subcortex of the frontotemporal and corona radiata, was found on the T2 FLAIR images (arrow). **d**-**f**: The DWI showed a symmetrically distributed strip-shaped hyperintensity of the corticomedullary junction in the bilateral frontal, parietal, and temporal lobes (arrow)

Encephalitis was initially suspected, and lumbar puncture was performed. The CSF examination showed that the CSF pressure was 110 mmH_2_O, and the white blood cell count was normal, but she had an increased total protein level of 0.57 g/l (normal 0.15–0.45 g/l) and a glucose level of 5.10 mmol/l (normal 2.2–3.9 mmol/l). The coxsackie B virus, enterovirus, and cytomegalovirus test levels were normal. Bacterial culture and *Cryptococcus neoformans* tests were negative. A contrast-enhanced MRI of the brain was normal. The diagnosis of encephalitis was excluded in general. However, leukoencephalopathy was evident on the T2 fluid-attenuated inversion recovery (FLAIR) images. A high-signal intensity in the white matter of the cerebral hemisphere, especially at the subcortex of the frontotemporal and corona radiata, was found on the T2 FLAIR images (Fig. 1a-c). The MRI imaging of the left basal ganglia and bilateral corona radiata showed lacunar infarction. Magnetic resonance angiography indicated that the blood vessels were normal. The DWI results revealed a symmetrically distributed strip-shaped high-intensity signal of the corticomedullary junction in the bilateral frontal, parietal, and temporal lobes (Fig. 1d-f).

**Fig. 2:**
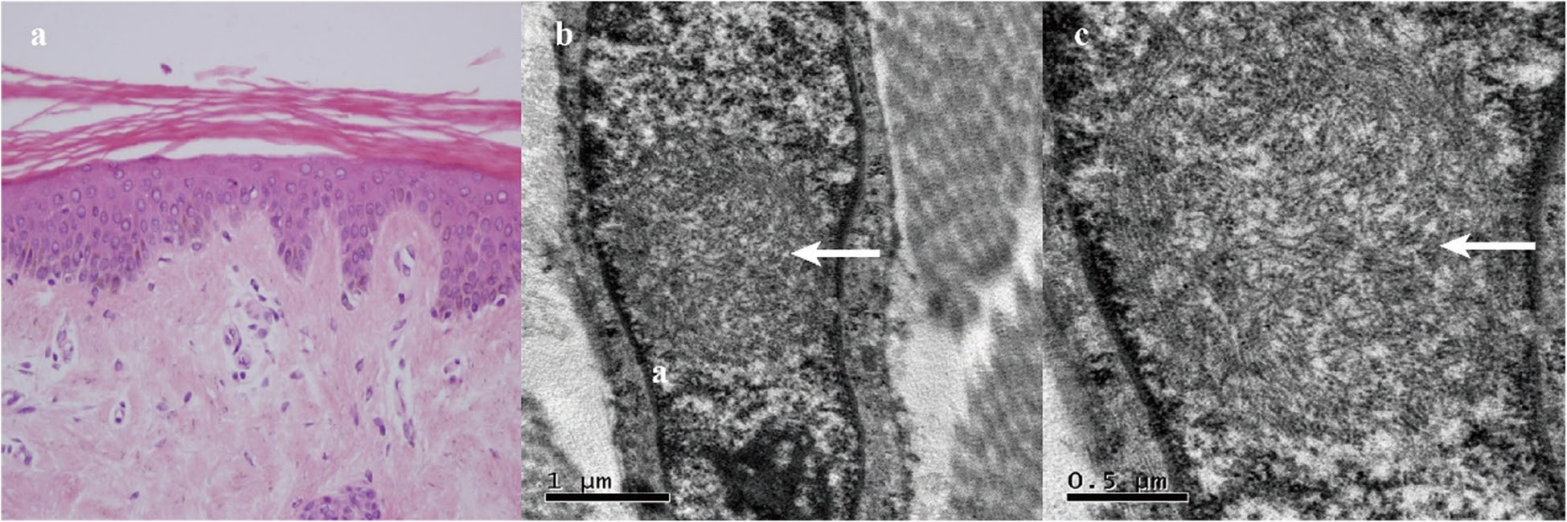
**a**-**c**: Skin biopsy performed 3 months after onset. **a**: Light microscopy showed hyperkeratosis of the epidermis. A few lymphocytes and tissue cells infiltrated through the superficial vessels of the dermis. **b** and **c**: Electron microscopy showed round-shaped intranuclear inclusions in the nucleus of the fibrocytes (arrow). The intranuclear inclusions had clear borders and were composed of fibrous substances without a membrane structure

Skin biopsy samples were obtained at 10 cm above the patient’s ankle. A light microscope was used to examine the samples, which showed hyperkeratosis of the epidermis. A few lymphocytes and tissue cells infiltrated through the superficial vessels of the dermis (Fig. 2a). Electron microscopy showed round-shaped intranuclear inclusions in the nucleus of the fibrocytes. The intranuclear inclusions had clear borders and were composed of fibrous substances without a membrane structure (Fig. 2b and c).

The fasting blood glucose was 9.48 mmol/l, and glycosylated hemoglobin was 6.6%. The routine urine test showed normal results. The patient presented with mental abnormality for 3 months. We did not consider the diagnoses of hypoglycemia, diabetic ketoacidosis, or hyperosmolar hyperglycemic syndrome. Furthermore, the patient did not present with depression, anxiety, consciousness disorders, hallucinations, and neurasthenia, which were common mental symptoms in patients with diabetes. The results of the electrolyte showed hypokalemia (2.6 mmol/L), hyponatremia (127.3 mmol/L), and hypochloremia (86.5 mmol/L). The renin-angiotensin-aldosterone system and cortisol hormone tests showed normal results. The MRI of the hypophysis showed no abnormal areas. The electroencephalography showed low-and moderate-amplitude desynchronized-mixed waves when she fell asleep.

Brain protection and symptomatic therapy were given when the patient was hospitalized, and the symptoms were relieved. However, she still complained of recurrent vomiting and urinary incontinence. The patient was alive, and the condition of the patient did not worsen or improve for 6 months after she was discharged.

## Discussion and conclusion

NIID is regarded as a heterogeneous disease. The most prominent initial symptom in sporadic NIID cases is dementia [7]. Miosis, ataxia, unconsciousness, abnormal behavior, encephalitic episode, bladder dysfunction, muscle weakness, and sensory disturbance are frequently observed. The patient mainly presented with fever, mental abnormality, cognitive impairment, and autonomic impairment characterized by vomiting and urinary incontinence. To the best of our knowledge, there are few cases of NIID with mental abnormality as the initial and main symptom in China [8]. Perhaps, they can be recognized as the mental abnormality prominent group. We did not consider the diagnosis of encephalitis owing to the normal body temperature, negative findings for viruses and autoimmune test in the serum samples, normal white-blood-cell count, bacterial culture, virus test, and *Cryptococcus neoformans* test in the CSF samples. The characteristic high-intensity signal in the corticomedullary junction on DWI, which is found in NIID cases diagnosed by skin biopsy or post-mortem autopsy, became a critical clue for diagnosing NIID [9]. We considered the diagnosis of NIID here. Sone et al. suggested that the intranuclear inclusions detected through skin biopsy were identical to those of central nervous cells for the ante-mortem diagnosis of sporadic NIID cases [7, 9]. Skin biopsy is useful for the antemortem diagnosis of NIID [4]. Therefore, skin biopsy was performed. Electron microscopy revealed that round-shaped intranuclear inclusions composed of fibrous substances were present in the nucleus of fibrocytes, which was consistent with the typical pathological changes in patients with NIID. In conclusion, we diagnosed the patient with a sporadic adult-onset NIID.

A strip-shaped high intensity signal of the corticomedullary junction on DWI, which might elucidate intranuclear inclusions in the brain, is a characteristic imaging feature of NIID. A high-intensity signal of the corticomedullary junction on the DWI was found in all sporadic NIID cases and extended along the corticomedullary junction as the disease progressed [7]. However, it was reported that the hyperintensities in the corticomedullary junction disappeared after several years [10, 11]. The pathological spongiotic changes of the NIID were related to subcortical DWI hyperintensities [12]. The DWI findings are likely to be dynamic at different stages of NIID, and it is essential to follow up on the patient with MRI changes.

A high-intensity signal in the middle cerebellar peduncles on T2-weighted imaging was a prominent feature in fragile X-associated tremor/ataxia syndrome (FXTAS) [13]. Nevertheless, leukoencephalopathy was obvious on T2-weighted image in NIID. In addition, an increased signal on T2 FLAIR images in the middle cerebellar peduncles was not found in this case. According to Wang et al., the significant DWI hypointensity in the thalamus and striatum was observed in male patients with FXTAS [14]. The characteristic high-intensity signal in the corticomedullary junction on DWI was found in the current case, which was different from FXTAS cases reported by Wang. We speculated that the strip-shaped high-intensity signal of the corticomedullary junction on DWI might be a good tool for distinguishing NIID from FXTAS. However, an abnormal high-intensity signal along the corticomedullary junction on DWI was found in three patients with FXTAS [15]. Notably, a high signal intensity in the paravermal area and abnormal high signal intensity in the middle cerebellar peduncles on T2 FLAIR images were found in patients with FXTAS, which was different from current case. In FXTAS, neuronal loss is only observed in the Purkinje cells, and intranuclear inclusions are not found in oligodendrocytes, which is different from NIID in pathological features [16]. Though there is a significant difference between FXTAS and NIID in post-mortem autopsy, the patient is alive and skin biopsy is useful and widely used for the antemortem diagnosis of NIID. As no study reported skin biopsy findings in FXTAS, it is difficult to exclude FXTAS by skin biopsy. It is necessary to evaluate DWI findings of the brain and skin biopsy findings in FXTAS in further studies. Currently, the FMR1 gene is useful for distinguishing NIID from FXTAS. Unfortunately, we did not detect the FMR1 gene in the patient owing to the patient’s refusal to allow us to perform the test. The possibility of FXTAS was not completely ruled out. The GGC repeat expansion at the 5′ end of NOTCH2NLC is associated with NIID and can be observed in patients clinically diagnosed with multiple system atrophy, Alzheimer’s disease, frontotemporal dementia, and parkinsonism [6, 17, 18]. Hence, it is important to perform genetic tests to further discover the molecular pathogenesis of the NIID.

In conclusion, we report a rare case of sporadic adult-onset NIID, which was diagnosed through a skin biopsy and typical MRI findings in China. The prevalence of NIID is likely to be higher than previously thought. Clinicians should raise the awareness of the diagnosis of NIID when a patient has mental abnormality and exhibits high-intensity signals on the DWI of the corticomedullary junction.

## Data Availability

All data generated or analyzed during this study can be obtained from the corresponding author on reasonable request.

## Abbreviations

NIID: Neuronal intranuclear inclusion disease
MRI: Magnetic resonance imaging
CSF: Cerebrospinal fluid
DWI: Diffusion-weighted imaging
CNS: Central nervous systems
MMSE: Mini-mental state examination
FLAIR: Fluid-attenuated inversion recovery
FXTAS: Fragile X-associated tremor/ataxia syndrome
